# Exploring the Utility of Cell-Free DNA Hydroxymethylation Profiling in Small-Cell Lung Cancer

**DOI:** 10.3390/ijms27104407

**Published:** 2026-05-15

**Authors:** Janice J. N. Li, Dangxiao Cheng, Luna J. Zhan, Danielle B. Sacdalan, Sami Ul Haq, Althaf Singhawansa, Vivek Philip, Natasha B. Leighl, Scott V. Bratman, Geoffrey Liu, Benjamin H. Lok

**Affiliations:** 1 Department of Medical Biophysics, Temerty Faculty of Medicine, University of Toronto, 101 College Street, Toronto, ON M5G 1L7, Canada; 2 Princess Margaret Cancer Centre, 610 University Ave, Toronto, ON M5G 2C4, Canada; 3 Institute of Medical Science, Temerty Faculty of Medicine, University of Toronto, 1 King’s College Circle, Toronto, ON M5S 1A8, Canada; 4 Schulich School of Medicine & Dentistry, Western University, London, ON N6A 5C1, Canada; 5 Radiation Medicine Program, Princess Margaret Cancer Centre, 610 University Ave, Toronto, ON M5G 2C4, Canada

**Keywords:** DNA hydroxymethylation, small-cell lung cancer, cell-free DNA, epigenetics, biomarkers

## Abstract

Small-cell lung cancer (SCLC) is an aggressive neuroendocrine carcinoma characterized by poor survival. Despite a high tumor mutation burden, biomarker discovery in SCLC remains challenging due to rapid tumor plasticity and limited tissue availability, highlighting the promise of liquid biopsy-based approaches. Epigenetic dysregulation of DNA 5-hydroxymethylcytosine (5hmC) has emerged as a cancer hallmark. However, its role in SCLC remains largely unexplored. Here, we characterized the cell-free DNA (cfDNA) 5hmC landscape in SCLC and evaluated its potential applications. We profiled the cell-free hydroxymethylomes of 107 pre-treatment SCLC patients and 53 matched controls using the 5hmC selective chemical labeling (5hmC-Seal) assay. SCLC displayed higher global 5hmC levels and distinct enrichment at neurodevelopmental and synaptic pathways, consistent with the neuroendocrine identity of SCLC. Concordance between plasma and matched circulating tumor cell patient-derived xenograft (CDX) demonstrated that cfDNA 5hmC reflects tumor epigenetic states and correlates with transcriptomic-derived molecular subtypes. Elevated SCLC-specific 5hmC levels and extensive stage (ES) disease were associated with inferior survival, with ES disease showing enrichment of pathways linked to cellular plasticity and neurodevelopment. Together, these findings indicate that cfDNA 5hmC profiling has potential as a biologically informative and clinically relevant biomarker in SCLC, with possible applications in tumor subtyping and risk stratification.

## 1. Introduction

Small-cell lung cancer (SCLC) is a deadly neuroendocrine carcinoma with a 5-year overall survival (OS) <8% [1]. At diagnosis, approximately two-thirds of patients present with extensive stage (ES) disease, which has a median OS of <10 months [2]. Identifying prognostic biomarkers for patient risk stratification and guiding therapeutic decisions is a critical unmet need.

Biomarker development in SCLC is hindered by the lack of actionable driver mutations, rapid tumor plasticity, and limited tumor tissue availability [3]. Although transcriptomic studies have identified four molecular subtypes (SCLC-A, -N, -P, -Y/I), each with distinct biology and treatment vulnerabilities, their clinical translation remains challenging [4,5]. These challenges highlight the need for minimally invasive approaches capable of capturing tumor biology in real time. In this context, liquid biopsy approaches offer a promising avenue for advancing biomarker discovery.

DNA 5-hydroxymethylcytosine (5hmC) is generated through the active DNA demethylation pathway. It is enriched in transcriptionally active regions and correlates with gene expression [6]. Importantly, 5hmC can be profiled in cell-free DNA (cfDNA), providing a minimally invasive way to assess gene regulatory programs. While many cancers exhibit global loss and local enrichment of 5hmC, its role in SCLC is poorly defined [7,8,9,10]. To date, only two small studies have examined 5hmC in SCLC, with 25 and 41 samples, respectively [11,12]. However, since these studies primarily focused on non-small-cell lung cancer (NSCLC), with SCLC included only as a secondary or validation cohort for their diagnostic models, the underlying role of 5hmC in SCLC is largely unknown. 

To address this gap, we present a comprehensive characterization of 5hmC in SCLC, providing novel insights into its biology and potential clinical utility. We show that 5hmC signatures can distinguish SCLC from non-cancer controls (NCC), reflect underlying molecular subtypes, and are associated with clinical outcomes. Together, these findings highlight the potential of 5hmC as a minimally invasive biomarker for SCLC classification and risk stratification.

## 2. Results

### 2.1. Cohort Overview and Assay Performance

**Figure 1:**
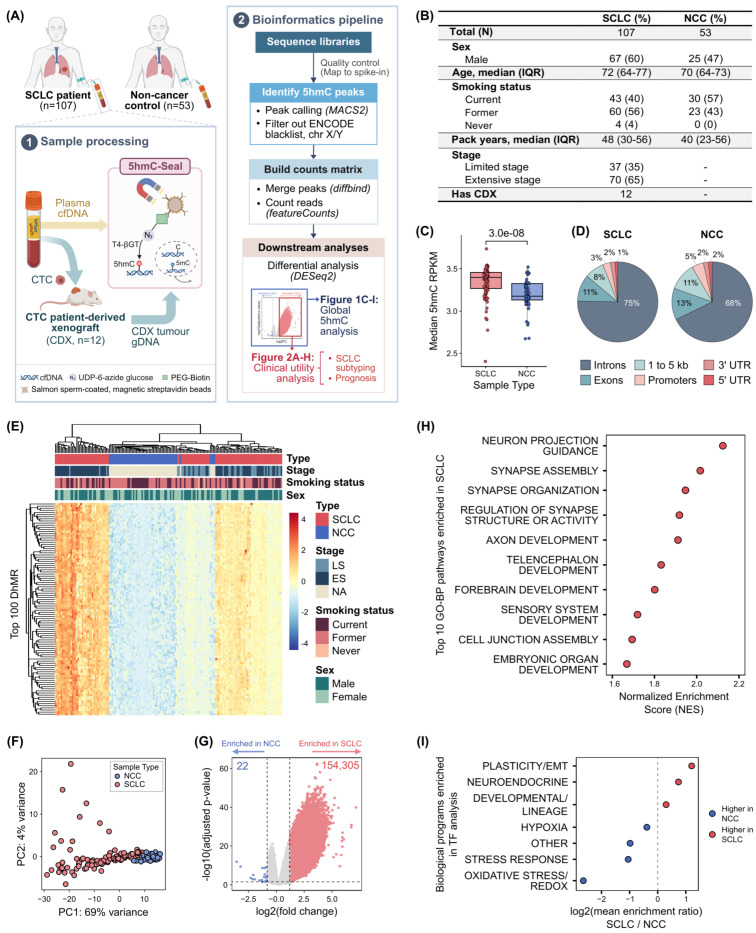
**Global 5hmC patterns can distinguish between SCLC and NCC.** (**A**) Study overview. (**B**) Patient demographics table. (**C**) Global 5hmC analysis between SCLC and NCC cfDNA samples. (**D**) Genomic distribution of 5hmC peaks across genic features. (**E**) Heatmap of the top 100 significant DhMR between SCLC and NCC by adjusted *p*-value. The color scale indicates the z-score, in which yellow/red represents medium/high 5hmC signals and blue represents low 5hmC signals, relative to the median. Each sample was annotated for sample type (SCLC vs. NCC), disease stage (LS, ES, and NA), smoking status (current, former, never), and sex (male, female). (**F**) Principal component analysis (PCA) of DhMR. (**G**) Volcano plot of DhMR between SCLC and NCC. Thresholds were set at |log_2_FC| > 1 and adjusted *p* < 0.05. (**H**) GSEA on DhMR enriched in SCLC vs. NCC using GO-BP terms. (**I**) Transcription factor (TF) motif analysis on DhMR between SCLC and NCC. TF motifs were categorized into biological programs. The log_2_ ratio of mean motif enrichment (SCLC/NCC) was calculated for each biological program. Positive values indicate higher enrichment in SCLC, while negative values indicate higher enrichment in NCC. 5hmC, 5-hydroxymethylcytosine; 5mC, 5-methylcytosine; C, cytosine; CDX, circulating tumor cell patient-derived xenograft; cfDNA, cell-free DNA; CTC, circulating tumor cell; DhMR, differentially hydroxymethylated region; EMT, epithelial–mesenchymal transition; ES, extensive stage; GO-BP, gene ontology–biological process; GSEA, gene set enrichment analysis; IQR, interquartile range; LS, limited stage; NA, not applicable; PC, principal component; REDOX, reduction–oxidation; RPKM, reads per kilobase per million mapped reads; T4-βGT, T4-beta glucosyltransferase; TF, transcription factor; UTR, untranslated region. Figure 1A was created in BioRender. Li, J. (2026) https://BioRender.com/l6nge3o (accessed on 8 May 2026).

We profiled plasma cfDNA from 107 pre-treatment SCLC patients and 53 NCC (Figure 1A). Demographic characteristics, including sex and smoking history, were comparable between groups (Figure 1B). Briefly, the SCLC cohort had a median age of 72 (interquartile range [IQR], 64–77), with 60% male, 96% smokers, and 65% diagnosed with ES disease.

To confirm the specificity of the Hydroxymethylation Selective Chemical Labeling (5hmC-Seal) assay, we evaluated the mapping recovery of 5hmC-amplified regions on spike-in controls (H-spike; Figure S1A). The 5hmC-Seal libraries demonstrated highly specific enrichment, with >99% on-target mapping to the H-spike (Figure S1B).

### 2.2. Global 5hmC Patterns Were Significantly Different in SCLC and NCC

Global 5hmC levels were significantly higher in SCLC than NCC, with median reads per kilobase of transcript per million mapped reads (RPKM) values of 3.4 (IQR, 3.27–3.46) and 3.18 (IQR, 3.13–3.33), respectively (Wilcoxon rank-sum test, *p* = 3.0 × 10^−8^; Figure 1C). SCLC and NCC shared a similar pattern of 5hmC distribution across genic regions, with most peaks being enriched in intronic regions (75% in SCLC vs. 68% in NCC; Figure 1D). However, the proportions differed significantly between groups (adjusted *p* < 0.001), except for the 3′ untranslated regions.

Principal component analysis (PCA) on all 667,000 5hmC peaks showed separation between SCLC and NCC, suggesting underlying global 5hmC differences (Figure S1C). Differential analysis (|log_2_FC| > 1, adjusted *p* < 0.05) identified 154,327 differentially hydroxymethylated regions (DhMR). Heatmap clustering revealed clear cohort separation, with SCLC exhibiting higher 5hmC levels (Figure 1E). PCA of these DhMR also showed distinct clustering, with NCC samples clustering tightly while SCLC samples were more dispersed (Figure 1F). Most DhMR were significantly hyperhydroxymethylated in SCLC (154,305; Figure 1G). Gene set enrichment analysis (GSEA) revealed strong enrichment for neurodevelopmental and synaptic pathways, aligning with the SCLC neuroendocrine identity (Figure 1H, Table S1). Motif enrichment analysis on the DhMR further revealed distinct transcription factor (TF) programs between SCLC and NCC (Figure 1I). SCLC showed enrichment of motifs related to neuroendocrine lineage, developmental/lineage specification, and cellular plasticity/epithelial–mesenchymal transition. In contrast, NCC was enriched for stress response, oxidative stress/reduction–oxidation homeostasis, and hypoxia-associated TF.

### 2.3. 5hmC Profiling Reveals Potential for SCLC Subtyping

**Figure 2:**
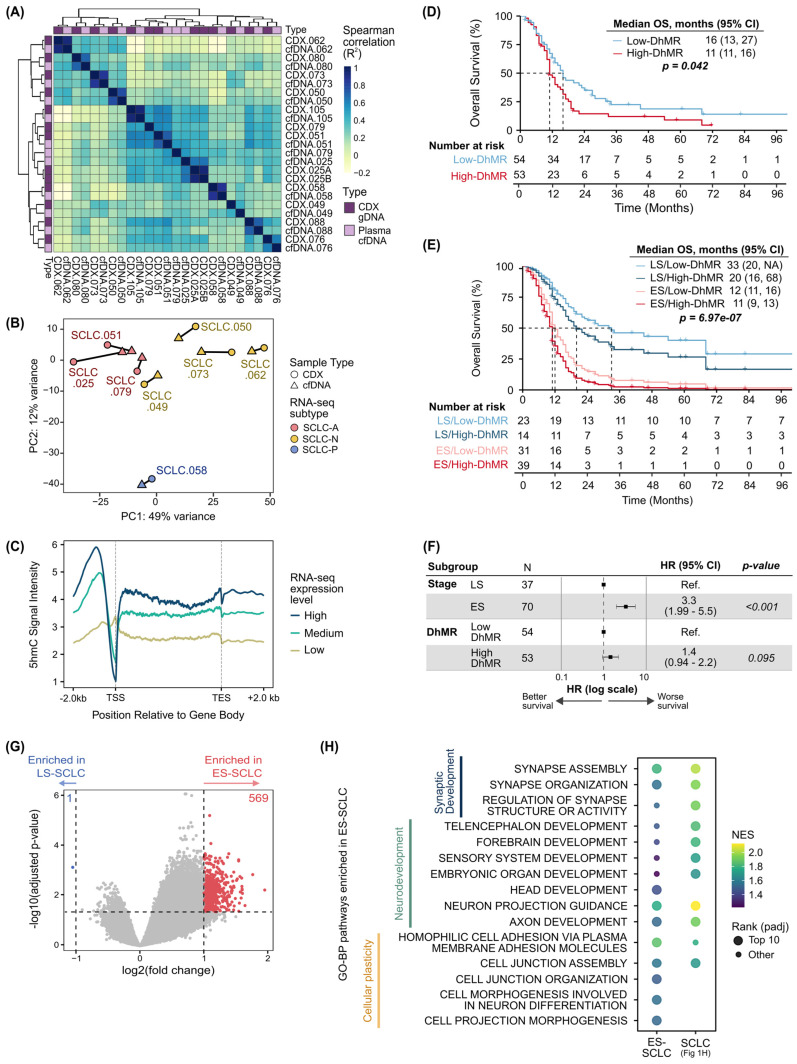
**Clinical utility of 5hmC as a molecular and prognostic biomarker.** (**A**) Correlation analysis between 5hmC patterns in matched plasma cfDNA and CDX gDNA. (**B**) PCA of DhMR in matched plasma cfDNA and CDX gDNA. Samples were annotated with their molecular subtype, derived from RNA-sequencing. (**C**) Metagene plot of 5hmC abundance by gene expression tertiles across the gene body. Kaplan–Meier overall survival analysis broken down by (**D**) global DhMR levels only or (**E**) stage and global DhMR levels. (**F**) Cox regression analysis of stage and 5hmC level as predictors of overall survival. (**G**) Volcano plot of DhMR between SCLC and NCC. Thresholds were set at |log_2_FC| > 1 and adjusted *p* < 0.05. (**H**) Comparison of top pathways identified by GSEA in ES-SCLC vs. LS-SCLC and SCLC vs. NCC. The dot color represents normalized enrichment score (NES) and the size denotes pathway rank by adjusted *p*-value (padj). CDX, circulating tumor cell patient-derived xenograft; cfDNA, cell-free DNA; DhMR, differentially hydroxymethylated region; ES, extensive stage; gDNA, genomic DNA; GO-BP, gene ontology–biological process; HR, hazard ratio; LS, limited stage; OS, overall survival; RNA-seq, RNA sequencing; TES, transcription end site; TSS, transcription start site.

We next assessed whether global 5hmC patterns reflected transcriptionally defined molecular subtypes. Matched CDX genomic DNA (gDNA) and patient plasma pairs showed high concordance in 5hmC signal across DhMR hyperhydroxymethylated in SCLC (SCLC-specific DhMR; Figure 2A). PCA using top DhMR also showed clustering of CDX-plasma pairs (Figure 2B). RNA-sequencing of CDX tumor tissue samples yielded sequencing quality metrics within acceptable ranges (Figure S1D). Overlaying RNA-sequencing-derived molecular subtypes revealed distinct subtype-specific clustering (Figure 2B and Figure S1D). Metagene analysis further showed a positive correlation between 5hmC abundance and gene expression, with the highest gene expression tertile displaying the highest 5hmC abundance (Figure 2C, Table S2). Together, these findings support cfDNA 5hmC as a potential surrogate for transcriptomic subtyping in SCLC.

### 2.4. Prognostic Value of 5hmC in SCLC

SCLC patients were stratified into low- and high-DhMR groups based on the summed, RPKM-normalized, SCLC-specific DhMR. Patients in the high-DhMR group had significantly worse OS than the low-DhMR group (median OS, 11 [95% CI: 11–16] vs. 16 [95% CI: 13–27] months, log-rank *p* = 0.042; Figure 2D). When disease stage was integrated, patients with ES-SCLC and high DhMR had the poorest survival (Figure 2E). Multivariable Cox regression revealed that stage was a significant factor (*p* = 4.21 × 10^−6^), while 5hmC was trending towards significance (*p* = 0.09; Figure 2F). To better understand the relationship between stage and 5hmC, we performed differential analysis by stage. More DhMR were hyperhydroxymethylated in ES-SCLC (569/154,305) than in limited stage-SCLC (1/154,305; Figure 2G), and mapped to pathways related to neurodevelopment, synaptic assembly, and cell morphogenesis (Figure 2H, Table S3).

## 3. Discussion

To our knowledge, this study represents one of the largest and most comprehensive investigations on the SCLC hydroxymethylome. Prior studies have been limited by small cohorts and often analyzed SCLC in combination with its non-small-cell counterpart [11,12]. Hu and colleagues (2022) included 25 SCLC samples, while Ren et al. (2023) had 41 SCLC patients [11,12]. In contrast, our SCLC-focused analysis included 107 SCLC samples and revealed distinct, biologically relevant 5hmC patterns associated with the SCLC neuroendocrine identity.

Across cancers, 5hmC has emerged as a cancer hallmark, reflecting its lineage state and epigenetic identity [6,9,13,14]. In prostate cancer, Sjöström et al. (2022) demonstrated that localized tumors exhibited a prostate-specific 5hmC pattern, whereas metastatic castration-resistant and transdifferentiated subtypes lost their prostate-associated 5hmC signatures and gained 5hmC signatures resembling those of their metastatic site [9]. Similarly, Wan et al. (2025) showed that 5hmC profiles could distinguish between prostate cancer subtypes [13]. Consistent with these observations, our study showed that DhMR that were more enriched in SCLC were preferentially associated with neuroendocrine pathways. Motif enrichment analysis on DhMR further supported these findings, showing enrichment of neuroendocrine, developmental, and plasticity-related TF programs in SCLC. Additionally, DhMR distinct in ES-SCLC were enriched in pathways involved in cell morphogenesis and cell junction organization, aligning with the disseminated nature of advanced SCLC. This suggests that 5hmC may have functional roles in reinforcing lineage-specific programs while simultaneously permitting adaptive cellular remodeling in ES disease. While additional validation is required, 5hmC has the potential to offer deeper insight into mechanisms associated with SCLC aggressiveness.

One of the most promising aspects of cfDNA 5hmC is its capacity to reflect tumor-specific epigenetic patterns. In our study, we observed strong concordance between 5hmC profiles from CDX gDNA and their matched patient plasma cfDNA, demonstrating that cfDNA is reflective of the underlying tumor hydroxymethylome. PCA further revealed molecular subtype-aligned clustering across CDX and cfDNA samples, indicating that 5hmC could capture patterns consistent with transcriptomic subtyping. Compared to other circulating biomarkers being investigated, like DNA methylation, fragmentomics, and nucleosome positioning, utilizing cell-free 5hmC for subtyping may be more advantageous as it preferentially marks transcriptionally active and lineage-defining regulatory elements [15,16,17]. Our metagene analysis further showed that 5hmC levels were positively associated with gene expression, supporting its relevance to transcriptional activity. This makes cfDNA 5hmC an attractive candidate for non-invasive transcriptomic subtyping of SCLC, addressing a major challenge in the field where tumor tissue is often limited.

Our prognostic analysis also supported the clinical relevance of 5hmC profiling. Higher SCLC-specific 5hmC levels were associated with worse OS, particularly in extensive-stage disease. Although stage remained the predominant predictor in Cox regression analysis, 5hmC approached significance, suggesting it may provide complementary prognostic value. This was not surprising, as disease stage is a prognostic biomarker in cancer [2,18]. Because our stratification relied on global 5hmC levels, it may capture 5hmC signals from both functional and non-functional regions. Future studies should explore whether biologically informative DhMR or machine learning-derived signatures can enhance the prognostic resolution.

In contrast to the commonly reported global loss of 5hmC in cancer, we observed global enrichment of 5hmC levels in SCLC relative to NCC [7,8,11,19]. However, this was not unprecedented, as 5hmC levels may vary by tumor type and study population [7,9,10]. Hu et al. (2022) reported global 5hmC gains in NSCLC, reasoning that 5hmC profiles may differ by ethnicity [11]. Song et al. (2017) noted elevated global 5hmC levels for hepatocellular carcinoma and glioblastoma, despite loss in lung cancer [7]. Elevated 5hmC may also reflect TET enzyme dysregulation driven by driver mutations. In hematologic malignancies, *MYC*-driven *TET1* increased 5hmC, while its inactivation resulted in genome-wide loss of 5hmC [20]. Given the established role of *MYC* in SCLC, a similar mechanism may warrant further investigation [21].

This study had several limitations. Firstly, some NCC and SCLC libraries were sequenced at different read depths. To account for technical variations, we down-sampled libraries, compared technical replicates for three pilot samples by PCA, and performed batch correction (Figure S1F–H). Secondly, we lacked matched tumor tissue for most cfDNA samples. As an alternative, we leveraged CDX models as they have been shown to recapitulate molecular and transcriptional features of donor patient tumors [22]. However, even with this approach, multi-omics integration and analyses were limited by the availability of matched RNA-sequencing data from CDX models (n = 12), reflecting known technical challenges in establishing these systems (~15–30% take rate) [23,24]. To avoid overinterpretation from a small paired dataset, we focused our analyses on a large plasma cohort (n = 107). This highlights a key strength of cfDNA-based approaches, which are more scalable and clinically accessible. We anticipate that the 5hmC profiles generated from our cohort of 107 plasma cfDNA samples and 12 CDX models will provide a valuable resource for the SCLC research community, particularly for future multi-omics integration efforts. As larger, prospectively collected datasets become available, integrative analyses will be important for validating and further refining the biological and clinical insights identified in our study.

## 4. Materials and Methods

### 4.1. Patient Recruitment and Sample Processing

All blood samples were collected between 2006 and 2023 upon written informed consent and approval by the institutional ethics committee and Research Ethics Board at the Princess Margaret Cancer Centre, Toronto, Canada. Only pre-treatment patients with de novo SCLC were included in this study. Healthy donors were age-matched, with smoking histories comparable to the SCLC cohort.

Two 10 mL EDTA tubes of blood were collected pre-treatment for each donor. Peripheral blood samples processing, cfDNA extraction, and circulating tumor cell patient-derived xenograft (CDX) generation were performed as previously described [25].

### 4.2. 5hmC-Seal Library Preparation

Samples were enriched for 5hmC using the 5hmC-Seal assay, with slight modifications [26]. Briefly, 10 ng of cfDNA spiked with 1 pg amplicons were end-repaired, 3′-adenylated, and ligated to xGen^TM^ UDI-UMI Adapters (Integrated DNA Technologies [IDT], cat. no. 10006914; Coralville, IA, USA) using the NEBNext^®^ Ultra^TM^ II DNA Library Prep Kit for Illumina^®^ (New England BioLabs [NEB], cat. no. E7645L; Ipswich, MA, USA). Libraries were then chemically labeled, biotinylated, and captured by Dynabeads^TM^ M-270 Streptavidin beads via the Huisgen cycloaddition (“Click”) chemistry reaction (Figure 1A). Libraries were amplified using the 2× Q5 master mix (NEB, cat. no. E7645L; Ipswich, MA, USA) and specially designed P5/P7 primers (P5: 5′-AATGATACGGCGACCACCGAGAT-3′; P7: 5′-CAAGCAGAAGACGGCATACGAGAT-3′), followed by purification with 0.9× Ampure beads. All libraries were quantified using the Qubit dsDNA High Sensitivity Assay (ThermoFisher, cat no. Q33231; Walham, MA, USA) and Bioanalyzer dsDNA High Sensitivity Assay (Agilent, cat no. 5067-4626; Santa Clara, CA, USA) prior to sequencing.

### 4.3. Spike-In Generation

Spike-in controls were prepared by combining equal amounts of C-spike, 5mC-spike, and 5hmC-spike in a 1:1:1 ratio, as previously described [26]. To generate these amplicons, lambda DNA (Thermo Fisher Scientific, cat. no. SD0021) was PCR amplified using unmodified (dCTP), methylated (dmCTP), or hydroxymethylated cytosine (10% dhmCTP with 90% dCTP), respectively, to create 3 sets of non-overlapping 190 bp sequences. The primer sequences used were as follows: dCTP: F-5′-TAAGGCGTTTCCGTTCTTCTT-3′, R-5′-GATACTCGCACCGAAAATGTC-3′; dmCTP: F-5′-CGGGTTATGATGAACTTGCTG-3′, R-5′-AGGCAACATGAAAACGCATAA-3′; and dhmCTP + 90%dCTP: F-5′-GGATGAAAACGAAAGGGGATA-3′, R-5′-GTCCAGCTGGGAGTCGATAC-3′. PCR products were then verified and purified by electrophoresis on a 2% (wt/vol) agarose gel, extracted from the gel, and quantified with the Qubit assay.

### 4.4. Sequencing and Data Processing

The 5hmC-Seal libraries were sequenced using the Illumina NovaSeq6000 or NovaSeqX instruments (Illumina; San Diego, CA, USA) according to the manufacturer’s instructions. All samples were sequenced with 150 bp paired-end reads at 50–80 million paired-end reads per sample. Reads were trimmed using TrimGalore! (version 0.5.0), aligned to the GRCh37/hg19 human genome (iGenomes, Illumina; San Diego, CA, USA) using BWA-MEM (version 0.7.15), and sorted, deduplicated, and indexed using SAMtools (version 1.12), as previously described [25]. Reads were also aligned to the spike-in controls for quality control (Figure S1A,B). The 5hmC peaks were called using MACS2 (version 2.1.2) in paired-end mode using default settings (*p*-value cut-off = 1 × 10^−5^). UCSC ENCODE blacklist regions and sex chromosomes were removed using Bedtools (version 2.27.1). Consensus peak sets were consolidated using DiffBind (version 3.6.5) and quantified with *featureCounts* in the subread package (version 2.0.1). Subsequent analyses were conducted in R (version 4.2.1). Genome-wide 5hmC regions were normalized with RPKM using edgeR (version 3.38.4) and annotated for basic genomic features using the annotatr R package (version 1.22.0).

RNA-sequencing libraries generated from CDX tumor tissue were sequenced using the Illumina NovaSeq6000 instrument at 100 million paired-end reads per sample. Reads were aligned to the hg19 reference genome (GENCODE) using STAR (version 2.7.9a). Quality control was performed by determining the percentage of uniquely mapped reads, reads unmapped (mismatch), reads mapped to multiple loci, and chimeric reads (Figure S1D). Each CDX was classified into a molecular subtype based on the TF (*ASCL1*, *NEUROD1*, *POU2F3*, and *YAP1*) exhibiting the highest normalized RNA expression, as previously described (Figure S1D) [4].

### 4.5. Differentially Hydroxymethylated Region (DhMR) Analysis

Differential analysis was performed using all 667,000 5hmC peaks identified in the consensus set between all pre-treatment SCLC and NCC cfDNA samples. DhMR were identified as peaks with |log_2_FC| > 1 and adjusted *p*-value < 0.05. Batch effects were corrected using ComBat-Seq from the sva package (version 3.44.0) in R. DhMR analysis was performed using DESeq2 (version 1.36.0) in R. Raw counts were normalized by median-of-ratios normalization, then gene-wise counts were modeled via negative binomial generalized linear models. Differential expression was tested using the Wald test and resulting *p*-values were adjusted to account for multiple testing. DhMR were visualized by heatmap (pheatmap, version 1.0.13) or by PCA, using the built-in *plotPCA* in the DESeq2 package or *prcomp* from the base R stats package.

### 4.6. 5hmC and RNA-Sequencing Correlation Analysis

The 5hmC profiles for 12 paired CDX genomic DNA (gDNA)-plasma cfDNA samples were compared using Spearman’s correlation in R. Only peak regions that overlapped in the cfDNA/CDX consensus peak set and the SCLC-specific DhMR were included in this analysis. Correlation coefficients were visualized with a heatmap.

Metagene profiles were generated using deepTools (version 3.5.2). The 5hmC signals were computed across gene bodies (±2 kb) for three gene expression tertiles (low, medium, and high) using *computeMatrix*. Profiles were subsequently averaged using *plotProfile* and visualized in R using ggplot2 (version 3.5.1).

### 4.7. Pathway Analysis

Gene set enrichment analysis (GSEA) was performed using the fgsea package (version 1.24.0) in R, with a focus on gene ontology–biological process (GO-BP) pathways. Genes were ranked based on their log_2_ fold change (log_2_FC) of DhMR and enrichment was assessed to identify relevant biological pathways.

### 4.8. Transcription Factor Motif Analysis

Motif enrichment analysis was performed on DhMR between SCLC and NCC derived by DESeq2. The *findMotifsGenome.pl* function in HOMER (version 5.1) was used with “hg19” as the reference genome and “-size given” to preserve peak widths. Known motif results were imported into R, and motif labels were cleaned and standardized. Enrichment was calculated as the ratio of target sequences versus background sequences. The top 50 enriched motifs per group (SCLC and NCC) were selected and mapped to TF families. Motifs were then grouped into biological programs based on the prior literature. Mean motif enrichment was calculated for each program. Program-level differences were summarized as the log_2_ ratio of the mean enrichment (SCLC/NCC), with a small pseudocount added to avoid division by zero.

### 4.9. Survival Analysis

The SCLC cohort was split into a high-DhMR and low-DhMR group by the median of summed RPKM in SCLC-specific DhMR. OS was calculated from the date of SCLC diagnosis to the date of death or last follow-up. Kaplan–Meier curves and log-rank test were performed using the survminer (version 0.5.0) and survival (version 3.8.3) packages in R. Cox proportional hazards regression was performed using the *coxph* function in the survival package. Hazard ratios were reported with 95% confidence intervals (CIs).

### 4.10. Statistical Analysis

All statistical analyses were performed using R (version 4.2.1). Clinical and demographic features were summarized descriptively. Statistical significance was defined as a *p*-value < 0.05.

## 5. Conclusions

In conclusion, this study demonstrated that cell-free 5hmC profiling captures tumor-specific features that are biologically relevant in SCLC, notably identifying pathways linked to neuroendocrine lineage and disease progression. These findings also support the potential utility of cfDNA 5hmC as a minimally invasive approach to complement existing strategies for patient risk stratification and to enhance the biological understanding of SCLC subtypes. While further validation is required, our work provides a large and well-characterized plasma-based dataset that serves as a valuable resource for future multi-omics studies in SCLC. Ongoing work should focus on region-specific 5hmC patterns, integration with other omics data, and exploring the role of 5hmC in therapeutic resistance and disease progression.

## Data Availability

De-identified SCLC, NCC, and CDX hydroxymethylation count matrices have been deposited to Zenodo (10.5281/zenodo.19216411) and are publicly available as of the publication date. RNA-seq CPM-normalized counts matrix and relevant clinical data have also been deposited to Zenodo. All original code has been deposited to GitHub (https://github.com/benloklab/5hmC_SCLC.git, accessed on 8 May 2026) and is publicly available as of the date of publication. Requests for raw fastq files and additional information should be directed to the corresponding author, Benjamin H. Lok (benjamin.lok@uhn.ca).

## Supplementary Materials

The following supporting information can be downloaded at: https://www.mdpi.com/article/10.3390/ijms27104407/s1.
