# Supplementary material for: Exploring the Utility of Cell-Free DNA Hydroxymethylation Profiling in Small-Cell Lung Cancer
Source: Int J Mol Sci. 2026 May 15;27(10):4407. doi: 10.3390/ijms27104407 (PMC13207598; doi:10.3390/ijms27104407)
Supplement: Supplementary file 1 [file ijms-27-04407-s001.zip › supplemental_figs_tables_final_14MAY2026.pdf]

Supplementary data

Figure S1. Quality control analyses of performed assays.
A) Overview of spike-in DNA generation. Lambda phage DNA was PCR amplified with nucleotides containing cytosine (C-spike), methylcytosine (M-spike), or a mixture of 90% cytosine and 10% 5hmC (H-spike) to create 3 sets of non-overlapping 190 bp sequences. B) 5hmC-Seal mapping efficiency to spike-in controls. The proportion of each spike-in (C-/M-/H-spike) was quantified and plotted as a fraction of the total reads mapped to the spike-in control. Error bars indicate standard deviation. C) Principal component analysis (PCA) prior to differential analysis. PCA showed separation between SCLC and NCC. 667,000 5hmC peaks were considered. D) Mapping efficiency of RNA sequencing samples. The percentage of reads uniquely mapped reads, reads unmapped due to mismatch, reads mapped to multiple loci, and chimeric reads were averaged across samples and plotted. Error bars indicate standard deviation. E) Classification of CDX tissue samples. SCLC subtypes were determined based on the SCLC transcription factor with the highest expression. F) RPKM-normalized global 5hmC levels at a sequencing depth of 50 million (M) reads. G) PCA of pilot samples sequenced at different read depths. H) PCA before batch correction. CDX, circulating tumour cell patient-derived xenograft; cfDNA, cell-free DNA; gDNA, genomic DNA; NCC, non-cancer control; QC, quality control; RPKM, reads per kilobase of transcript per million mapped reads; SCLC, small cell lung cancer. Figure S1A was created in BioRender. Li, J. (2026) https://BioRender.com/l6nge3o (accessed on 14 May 2026).

Supplementary tables

Table S1. GSEA results for SCLC vs NCC DhMRs

Table S2. 5hmC peaks and their associated gene expression in SCLC CDX samples

Table S3. GSEA results for ES- vs LS-SCLC DhMRs

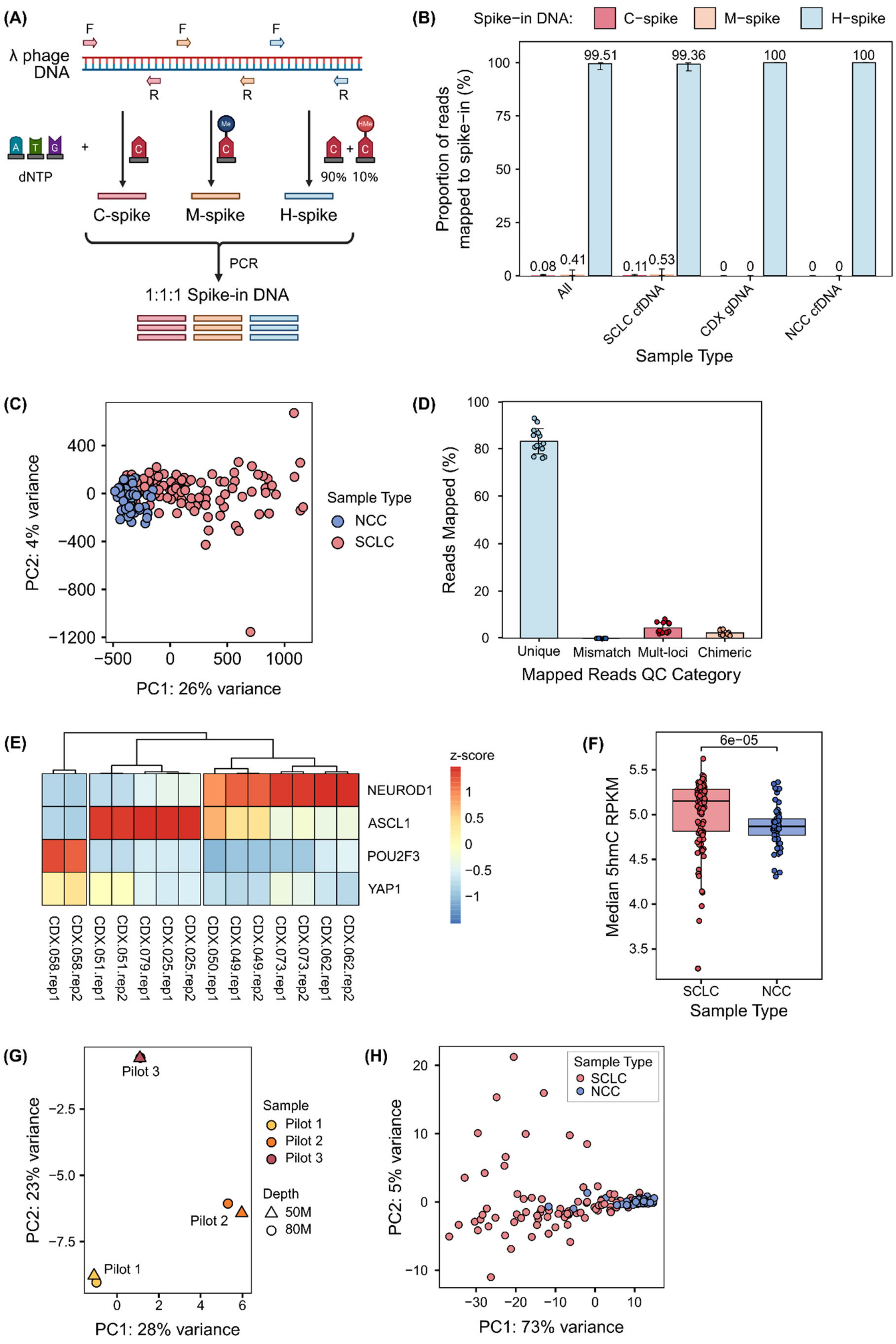

(A)
λ phage DNA
F
R
dNTP
90% 10%
C-spike
M-spike
H-spike
PCR
1:1:1 Spike-in DNA
(B)
Spike−in DNA: C−spike M−spike H−spike
Proportion of reads mapped to spike−in (%)
0.08 0.41 99.51
0.11 0.53 99.36
0 0 100
0 0 100
All
SCLC cfDNA
CDX gDNA
NCC cfDNA
Sample Type
(C)
PC2: 4% variance
PC1: 26% variance
Sample Type
NCC
SCLC
(D)
Reads Mapped (%)
Unique
Mismatch
Mult-loci
Chimeric
Mapped Reads QC Category
(E)
NEUROD1
ASCL1
POU2F3
YAP1
z-score
CDX.058.rep1
CDX.058.rep2
CDX.051.rep1
CDX.051.rep2
CDX.079.rep1
CDX.025.rep1
CDX.025.rep2
CDX.050.rep1
CDX.049.rep1
CDX.049.rep2
CDX.073.rep1
CDX.073.rep2
CDX.062.rep1
CDX.062.rep2
(F)
6e−05
Median 5hmC RPKM
SCLC
NCC
Sample Type
(G)
Pilot 3
Pilot 2
Pilot 1
PC2: 23% variance
PC1: 28% variance
Sample
Pilot 1
Pilot 2
Pilot 3
Depth
50M
80M
(H)
Sample Type
SCLC
NCC
PC2: 5% variance
PC1: 73% variance
